# Occurrence of Sensorineural Hearing Loss in Viral Meningitis and Encephalitis

**DOI:** 10.1002/jmv.71171

**Published:** 2026-10-05

**Authors:** Elisa Skovgaard Jensen, Andreas Vestergaard Jensen, Per Cayé‐Thomasen, Pelle Trier Petersen, Birgitte Rønde Hansen, Nichlas Hovmand, Rannvá Winther, Jonas Bruun Kjærsgaard, Malene Kirchmann, Christian T. Brandt, DASGIB Study Group, Helene Mens, Birgitte Rønde Hansen, Hans Rudolf Lüttichau, Micha Jepsen, Christian Brandt, Christian Østergaard Andersen, Lykke Larsen, Merete Storgaard, Jacob Bodilsen, Henrik Nielsen

**Affiliations:** ^1^ Department of Otorhinolaryngology Nordsjællands Hospital, University of Copenhagen Hillerød Denmark; ^2^ Department of Otorhinolaryngology Head and Neck Surgery and Audiology, Rigshospitalet University Hospital of Copenhagen Copenhagen Denmark; ^3^ Department of Infectious Diseases Zealand University Hospital, University of Copenhagen Roskilde Denmark; ^4^ Faculty of Health and Medical Sciences University of Copenhagen Copenhagen Denmark; ^5^ Department of Infectious Diseases, Hvidovre Hospital University of Copenhagen Hvidovre Denmark; ^6^ Department of Infectious Diseases, Aalborg University Hospital University of Aalborg Aalborg Denmark; ^7^ Department of Otorhinolaryngology Head and Neck Surgery and Audiology Aalborg University Hospital, University of Aalborg Aalborg Denmark; ^8^ Department of Clinical Medicine Aalborg University Aalborg Denmark; ^9^ Department of Infectious Diseases, Rigshospitalet University Hospital of Copenhagen Copenhagen Denmark; ^10^ Department of Infectious Diseases Herlev Hospital, University of Copenhagen Denmark; ^11^ Department of Infectious Diseases Nordsjællands Hospital, University of Copenhagen Hvidovre Denmark; ^12^ Statens Serum Institut (SSI) Copenhagen Denmark; ^13^ Department of Infectious Diseases Odense University Hospital, University of Southern Denmark Odense Denmark; ^14^ Department of Infectious Diseases Aarhus University Hospital, University of Aarhus Aarhus Denmark

**Keywords:** encephalitis, hearing loss, otoacoustic emissions, viral meningitis

## Abstract

Hearing loss in viral neuroinfections is poorly investigated. The present study aimed to clarify if hearing loss can develop in viral meningitis and encephalitis. An OAE (*oto‐acoustic emission)* was measured in adult patients with viral meningitis and encephalitis on the day of admission and 30–60 days after discharge. Frequencies were categorized as Low (1, 1.5, 2 kHz), Mid (3, 4, 5 kHz), Mid‐high (6, 7, 8 kHz), and High (9, 10 kHz). An OAE was measured in 41 patients with viral meningitis and 8 patients with encephalitis. Six patients with viral meningitis (15%) experienced a decrease of > 5 dB in one or more frequency categories. Twelve patients (29%) had a temporary decrease in OAE levels of more than 5 dB and 23 patients (54%) had no change in OAE levels between admission and follow‐up. We found no significant differences in OAE levels from admission to follow‐up between viral meningitis pathogens. In viral encephalitis, herpes simplex virus was associated with a decrease in OAE levels in all frequencies in 3 of 5 patients. Varicella zoster encephalitis was not associated with changes in OAE levels. Viral meningitis and herpes simplex encephalitis is associated with development of a sensorineural hearing loss.

## 1 Introduction

Viral meningitis and viral encephalitis in adults are not generally perceived to be associated with any risk of sensorineural hearing loss in contrast to the extensive hearing loss occurring in bacterial meningitis [1, 2].

However, hearing loss is among the most common sequelae due to West Nile virus infections [3]. Also, congenital sensorineural hearing loss due to Cytomegalovirus is likely to be the most common cause of acquired sensorineural hearing loss where also Rubella, mumps and herpes simplex 1 and 2 are described as congenital viruses with otologic sequelae [4]. Finally, among children, the early presence of a herpes simplex virus antibody response is associated with hearing loss and the hearing loss therefore suspected to originate from infection with these viruses [5].

Our primary aim was therefore to investigate occurrence of sensorineural hearing loss in an unselected adult population admitted with a diagnosis of viral meningitis or viral encephalitis. We used distortion product otoacoustic emissions (OAE) to determine sensorineural hearing loss and eventual recovery from a sensorineural hearing loss [1, 6].

## 2 Materials and Methods

### 2.1 Study Design

This was a prospective, observational study in Denmark. Patients were included at the Department of Infectious Diseases at one university hospital from December 2017 to February 2020 (Nordsjællands Hospital, University of Copenhagen) and two university hospitals from January 2019 to January 2020 (Aalborg University Hospital and University Hospital Copenhagen Hvidovre).

### 2.2 Setting and Study Population

Adults aged ≥ 18 years admitted on the suspicion of viral meningitis or viral encephalitis were screened for inclusion.

*Inclusion criteria—Viral meningitis* [7]. A clinical presentation consistent with viral meningitis combined with either: 1) Detected viral DNA/RNA in the cerebrospinal fluid (CSF), 2) CSF leukocytes ≥ 10 cells/µL and positive intrathecal synthesis of antibodies specific for herpes simplex virus (HSV‐1 or HSV‐2) or varicella zoster virus (VZV), 3) CSF leukocytes ≥ 10 cells/µL and detected viral DNA/RNA from skin lesions or serology suggestive of an acute infection with a neurotropic virus, or 4) CSF leukocytes ≥ 10 × 10^6^ cells/L and no other diagnosis considered more likely at last follow‐up.

*Inclusion criteria—Viral encephalitis* [7]. A clinical presentation suggestive of encephalitis, e.g., impaired consciousness and mental changes for more than 24 h, headache, neurological deficit, and seizures combined with either 1) Detected viral DNA/RNA in the CSF, 2) CSF leukocytes ≥ 10 cells/µL and positive intrathecal synthesis of antibodies specific for HSV‐1, HSV‐2, or VZV, 3) CSF leukocytes ≥ 10 cells/µL and detected viral DNA/RNA from skin lesions or serology suggestive of an acute infection with a neurotropic virus or 4) CSF leukocytes ≥ 10 cells/µL and/or CNS imaging suggestive of encephalitis, and no other diagnosis considered more likely given all available information.

*Exclusion criteria*. Pre‐existing hearing loss, head trauma, significant history of noise exposure, ear surgery, concurrent or previous treatment with ototoxic agents (e.g., gentamycin), prior CNS pathology, and a history of prior CNS infections.

### 2.3 Distortion Product Otacoustic Emissions

An OAE were recorded using the Interacoustics Titan DPOAE 440 module. Eleven frequencies were measured in each ear: 1 kHz, 1.5 kHz, 2 kHz, 3 kHz, 4 kHz, 5 kHz, 6 kHz, 7 kHz, 8 kHz, 9 kHz, and 10 kHz. The frequency ratio (f2/f1) was fixed at 1.22. OAE was measured bedside with patients lying down on their backs with a 30° head position. OAE levels were registered for each ear. Otoscopy and tympanometry preceded each measurement; in cases of outer or middle ear pathology, the affected ear was excluded.

Frequencies were categorized as low (1, 1.5, 2 kHz), mid (3, 4, 5 kHz), mid‐high (6, 7, 8 kHz), and high (9, 10 kHz).

An OAE measurement were performed at:

1. Admission: Within 24 h from diagnostic lumbar puncture and initiation of treatment.
2. Between admission and day 3.
3. Follow up in the outpatient clinic 30 to 60 days after discharge.

### 2.4 Noise Floor

The noise floor was the average noise measured in department wards and outpatient clinics as previously described [1]. A signal‐to‐noise ratio of +3 dB was applied to the noise floor at low to mid‐high frequencies and +6 dB at high frequencies [8, 9]. Thus, the border of detection of an OAE within each frequency category was: Low −10 dB, Mid −15 dB, Mid‐high −13 dB, High −16 dB.

*OAE healthy control group*. OAE were obtained in 158 healthy persons with an even sex distribution divided into decennials from 18 to 80 + , as previously described [1]. Data are presented in Supporting Information Figure S1, showing the mean emission threshold levels among age‐ and sex‐matched healthy adults as comparator.

### 2.5 Statistical Evaluation

Data analysis and graphical presentations were performed using GraphPad PRISM v. 9.0 and R studio version 1.4.1717. Mann‐Whitney U test and Kruskal‐Wallis test was used to compare groups. Statistical significance was set at *p* < 0.05.

### 2.6 Primary Outcome and Data Handling

*Outcome*. An increase or decrease in OAE levels of > 5.0 dB between admission and follow‐up in one or more frequency categories was considered significant [10]. This was calculated as the difference between the patient's own OAE level on admission and at follow‐up.

*Data handling*. OAE levels are presented as the mean OAE level in dB of both ears within each frequency category. The 11 frequencies were categorized into 4 frequency categories (low‐, mid‐, mid‐high‐ and high) where the average changes measured within each frequency category were calculated. There were no significant differences in OAE levels between the right and left ears except for patients presenting both with meningitis and the Ramsay Hunt syndrome. In these patients, OAE levels from right and left ear are presented separately due to significant difference in OAE levels between the paralytic and nonparalytic side.

## 3 Results

Viral meningitis, Tables 1 and 2 (Supporting Information Figure S1).

**Table 1:** Clinical characteristics in 41 patients with viral meningitis and 8 patients with viral encephalitis. Data shown as medians with interquartile range. Numbers with percentage in parenthesis.

Admission characteristics	Viral meningitis (*n* = 41)	Viral encephalitis (*n* = 8)
**Demographics**		
Age, years	37 (27–54)	58 (47–75)
Sex, male	19 (46)	3 (38%)
Duration of symptoms (days)	3 (1–6)	4 (3–10)
**Clinical characteristics**		
Headache	40 (98%)	3 (38%)
Photo‐ and phonofobia	25 (61%)	1 (25%), *n* = 4
Neck stiffness	13 (32%)	0
Temperature (°Celsius)	37.9 (37.5–38.6)	37.8 (37.4–37.9)
GCS ‐ Glasgow Coma Score	15 (15)	14 (11–15)
**Blood and CSF biochemistry**		
Blood leukocyte count, cells x 10^9^/L	8 (5.9–9.8)	4.9 (3.3–5.8)
C‐reactive protein, mg/L	6 (3–18)	5 (3–12)
CSF leukocyte count, cells x 10^6^/L	201 (83–321)	215 (27–374)
CSF protein, g/L	0.78 (0.53–1.1)	0.9 (0.48–1.6)
**Etiology**		
Enterovirus	15 (37%)	0
Herpes simplex‐2	6 (15%)	1 (12%)
Herpes simplex‐1	0	4 (50%)
Varizella Zoster	12 (29%)	3 (38%)
Other	2 (5%)	0
Unknown	6 (15%)	0
**Treatment**		
Aciclovir/Valaciclovir	15 (37%)	8 (100%)
Treatment duration	8 (4–14)	14 (14–21)
Dexamethasone	17 (41%)	0
No. of dexamethasone doses	2 (1–7)	0

In total, 68 patients with viral meningitis were eligible for inclusion of which 41 patients were available for analysis of hearing loss from admission to follow‐up. Twenty‐seven patients with viral meningitis were excluded of which one patient had previous HSV‐2 meningitis, one patient had pre‐existing hearing loss and 25 had no available follow‐up OAE.

Age was significantly lower in patients with enterovirus meningitis (median 28 years, IQR 25–35) and viral meningitis with unidentified pathogen (median 29 years, IQR 23–42) compared to VZV meningitis (median 50, IQR 42–57), Mann‐Whitney test *p* = 0.0001 and *p* = 0.031, respectively, but not HSV meningitis (median 51 years, IQR 26–67) and other viral pathogens (median 48, IQR 36–60), Mann‐Whitney test, *p* = 0.09 and *p* = 0.07, respectively.

### 3.1 OAE From Admission to Follow‐Up > 30 Days, Table 2

**Table 2:** Viral meningitis. OAE levels from admission to follow up. Data shown as medians with full range (min/max) in each frequency category. Only the number of patients with an increase or decrease in OAE levels in dB in each frequency category are shown. If fewer than three patients had an OAE change all data are shown. An increase or decrease in OAE levels were calculated as the difference in each patient's OAE levels in the designated time interval.

Viral meningitis			Change in OAE levels in dB with no. of patients in each category			
	Study interval ‐ Days	***n***	Low frequency 1, 1.5, 2 kHz	Mid frequency 3, 4, 5 kHz	Mid‐high frequency 6, 7, 8 kHz	High frequency 9, 10 kHz
Patients with **OAE level increase**						
All patients	1–3	6 of 33 (18%)	15.0 *n* = 1	11.7 and 12.2 *n* = 2	7.2 (6.1–14.7) *n* = 5	5.8 (5.1–10.0) *n* = 3
	1 to > 30	12 of 41 (29%)	6.3 (5.2–12.6) *n* = 4	8.8 (5.8–15.8) *n* = 5	7.2 (6.1–9.6) *n* = 3	8.2 (6.6–11.3) *n* = 3
Enterovirus	1 to > 30	3 of 15 (20%)	6.5 *n* = 1	15.8 *n* = 1	0	11.3 *n* = 1
HSV‐2	1 to > 30	3 of 6 (50%)	12.6 *n* = 1	13.3 *n* = 1	7.2 *n* = 1	0
VZV without Ramsay Hunt	1 to > 30	4 of 9 (44%)	5.2 and 6.1 *n* = 2	5.8 *n* = 1	9.6 *n* = 1	6.6 and 8.2 *n* = 2
VZV, Ramsay Hunt ‐ paralytic side	1 to > 30	2 of 3 (66%)	0	12.7 and 13.3 *n* = 2	7.1 and 8.6 *n* = 2	0
VZV, Ramsay Hunt ‐ nonparalytic side	1 to > 30	2 of 3 (66%)	0	8.1 *n* = 1	0	7.2 *n* = 1
Patients with **OAE level decrease**						
All patients	1–3	6 of 33 (18%)	−6.3 *n* = 1	−6.8 (−5.2 to −13.5) *n* = 3	−11.1 and −6.2 *n* = 2	−5.1 *n* = 1
	1 to > 30	6 of 41 (15%)	−16.9 and −9.3 *n* = 2	−13.2 (−18.0 to −12.2) *n* = 5	−6.9 *n* = 1	−8.7 and −5.2 *n* = 2
Enterovirus	1 to > 30	3 of 15 (20%)	−9.3 *n* = 1	−13.5 (−18.0 to −12.9) *n* = 3	−6.9 *n* = 1	0
VZV without Ramsay Hunt	1 to > 30	1 of 9 (11%)	0	−13.2 *n* = 1	0	0
Unknown pathogen	1 to > 30	2 of 6 (33%)	−16.9 *n* = 1	−12.2 *n* = 1	0	−8.7 and −5.2 *n* = 2

A decrease of > 5 dB in any frequency category was observed in 6 of 41 patients (15%). Twelve patients (29%) had increase of OAE levels > 5 dB between days 1 (admission) and 30 (follow up), and 23 patients (56%) had no change in OAE levels between admission and follow‐up.

*Viral pathogen*. There were no significant differences in OAE levels between pathogens in any frequency area between admission and follow‐up (Low‐, mid‐, mid‐high, and high; Kruskal‐Wallis Test, *p* = 0.8, *p* = 0.58, *p* = 0.18 and *p* = 0.06, respectively.

*Sex differences*. There were no significant differences between females and male in OAE levels between admission and follow‐up in any frequency area (Low‐, mid‐, mid‐high, and high; Mann‐Whitney test, *p* = 0.2, *p* = 0.58, *p* = 0.88 and *p* = 0.41, respectively.

*Enterovirus*, Table 2. A decrease in OAE levels was observed in 3 of 15 patients with all 3 experiencing decreases in the mid‐frequency area and 1 in the low‐ and mid‐high frequency area. An increase was observed in 3 patients in the low‐, mid‐ and high frequency area.

*Herpes simplex virus—HSV*, Table 2. Increase in OAE levels was observed in 3 patients in the low‐, mid‐ and mid‐high frequency area respectively. Three patients had unaffected OAE levels.

*Varicella zoster virus—VZV*, Table 2. A decrease in OAE levels was observed in 1 of 9 patients in the mid‐frequency area. An increase in OAE level was observed in 4 patients in the Low‐ mid‐, mid‐high and high frequency area.

*Varicella zoster virus—VZV with Ramsay Hunt syndrome*, Table 2. Two of 3 patients had increase of OAE levels on the paralytic side to the same OAE level increase on the nonparalytic side in the mid‐ and high frequency area respectively. One patient had OAE levels within the noise floor on the paralytic side and no increase at follow up.

*Other viruses (Tick borne encephalitis and Toscana sandfly virus)*, Table 2. OAE levels were unaffected from admission to follow‐up.

*Viral meningitis with unidentified pathogen*, Table 2. An OAE level decrease was observed in 2 of 6 patients in the high‐frequency area and one of these patients also had OAE level decrease the low‐ and mid‐frequency area. Four patients had no changes in OAE levels between admission and follow‐up.

Encephalitis, Tables 1 and 3.

**Table 3:** Viral encephalitis. OAE levels from admission to follow up. Data shown as medians with full range (min/max) in each frequency category. Only the number of patients with an increase or decrease in OAE levels in dB in each frequency category are shown. If fewer than three patients had an OAE change all data are shown. An increase or decrease in OAE levels were calculated as the difference in each patient's OAE levels in the designated time interval.

Viral encephalitis			Change in emission threshold levels (ETL) in decibel–dB			
	**Study interval–**Days	*n*	Low frequency 1, 1.5, 2 kHz	Mid frequency 3, 4, 5 kHz	Mid‐high frequency 6, 7, 8 kHz	High frequency 9, 10 kHz
Patients with **OAE level increase**						
All patients	1 to > 30	4 of 8 (50%)	6.1 *n* = 1	6.3 and 6.9 *n* = 2	0	8.2 *n* = 1
HSV‐1	1 to > 30	3 of 5 (60%)	0	6.3 and 6.9 *n* = 2	0	8.2 *n* = 1
VZV	1 to > 30	1 of 3 (33%)	6.1 *n* = 1	0	0	0
Patients with **OAE level decrease**						
All patients	1 to > 30	4 of 8 (50%)	0	−8.4 *n* = 1	0	0
HSV‐1	1 to > 30	3 of 5 (60%)	0	−8.4 *n* = 1	0	0
VZV	1 to > 30	1 of 3 (33%)	0	0	0	0

Twelve patients with viral encephalitis were eligible for inclusion of which 8 patients were available for analysis of an OAE from admission to follow up. Three patients with VZV encephalitis were excluded due to pre‐existing hearing loss and 1 patient had no available OAE at follow up. Median age was 47 years (IQR 46–63) for HSV encephalitis and 78 years (IQR 53–82) for VZV encephalitis. Age was not different between pathogens, Mann‐Whitney test, *p* = 0.13. Patient demographics are presented in Table 1.

*Herpes simplex virus encephalitis—HSV*. Of 5 patients with HSV encephalitis, 3 patients presented on admission with a low or absent OAE in all frequencies. Two patients remained with OAE levels below detection, close to the noise floor. A mid‐frequency decrease was observed in 1 patient and increase in 2.

*Varicella zoster encephalitis—VZV*. One patient had increase in OAE levels in the low frequency area. Two patients were unaffected from admission to follow up.

### 3.2 Treatment

*Aciclovir treatment*. Fifteen of 18 patients (83%) with HSV‐2 and VZV meningitis were treated with initial intravenous (i.v.) acyclovir for > 1 day of which 9 were switched to oral therapy after median 4 days (median treatment 10 days for i.v. aciclovir and oral valaciclovir combined). Two patients with HSV‐2 meningitis were not treated with acyclovir. Both patients had increase in OAE levels suggesting full recovery.

There was no difference in OAE level changes from admission to follow up comparing patients treated with acyclovir for a median of 10 days (*n* = 12) to patients treated for 1 day or not receiving treatment (*n* = 3), Mann‐Whitney test, *p* = 0.73, *p* = 0.65, *p* = 0.99 and *p* = 0.53 in the low‐, mid‐, mid‐high, and high frequency area respectively. All patients with viral encephalitis were treated with i.v. acyclovir for at least 14 days.

*Dexamethasone treatment*. Seventeen patients with viral meningitis received initial i.v. dexamethasone 10 mg four times daily as part of treatment regimen for suspected bacterial meningitis. Of these, seven patients received 1 dose, and 5 patients received 4 doses or more. Three patients with VZV meningitis and Ramsay‐Hunt syndrome were switched to oral prednisolone after 1 and 3 days respectively. There was no difference in OAE level changes from admission to follow‐up comparing untreated patients to patients treated with 1 dose or more of dexamethasone or the group receiving > 4 doses, Mann‐Whitney test, *p* = 0.58 and *p* = 0.11, respectively.

No patients with viral encephalitis were treated with dexamethasone.

## 4 Discussion

To our knowledge, the present study is the first to measure cochlear function using highly sensitive OAE in patients with viral meningitis and encephalitis and the first to document the association with sensorineural hearing loss which is the result of decreased OAE levels.

We found a decrease in OAE levels between admission and follow‐up in 15% of the patients with viral meningitis and a temporary decrease reflected by the subsequent increase in 29%. A marked decrease in OAE levels reflecting cochlear function was observed in 4 male patients of which three cases was due to enterovirus and one case with unidentified pathogen. An increase in OAE levels between admission and follow up was observed in meningitis due to HSV‐2 and VZV. Also, in cases not receiving acyclovir treatment. Patients with Ramsay Hunt syndrome treated with acyclovir or acyclovir with systemic steroids presented on admission with a significant decrease in OAE levels on the paralytic side but with subsequent increase to the level on the nonparalytic side in 2 of 3 patients. In the group of patients receiving 1 or more doses of dexamethasone until a suspicion of bacterial meningitis was ruled out, we found no difference between treated and untreated patients [11]. We would have expected some effect of this anti‐inflammatory treatment, but the outcome of HSV‐2 and VZV meningitis is good even the few cases not receiving treatment. Also, the pathogenesis of hearing loss as discussed below may not be primarily inflammatory induced, but also caused by direct viral invasion of cochlear hair cells [3, 4].

Compared to our previous studies in patients with bacterial meningitis and Lyme neuroborreliosis, cochlear function was affected to a lesser extent in viral meningitis [1, 6]. The pathogenesis and pathology are likely even more complex in viral infections involving both cochlear inflammation and direct viral invasion of hair cells. The latter being the proposed cause of congenital hearing loss [4, 12, 13, 14]. A marked decrease in OAE levels and thus cochlear function was observed in patients with HSV encephalitis. Four patients had affected cochlear function in all frequencies of which 3 had undetectable OAE levels. The common feature in these 4 patients was temporal lobe inflammation and edema whereas the last patient diagnosed with HSV encephalitis did not have any detectable brain pathology on brain imaging with MRI. We speculate that the pathogenesis in this situation may be similar to congenital hearing loss due to Herpes viruses [5, 12, 15]. Patients with a diagnosis of VZV encephalitis were older, and half the patients were excluded due to pre‐existing age‐related hearing loss. This reduced the spectrum of detection of any cochlear dysfunctions just due to age related decline in the ability of OAE to detect thresholds [1].

The relationship between facial nerve paralysis and hearing loss remains poorly understood. In a previous study of Lyme neuroborreliosis, we found no association between the site of facial nerve paralysis and cochlear function [6]. This contrasts with our findings in patients with Ramsay Hunt syndrome caused by VZV. In these patients, the emission threshold level on the paralytic side was impaired at admission but showed substantial recovery at follow‐up. Our findings are in line with a previous study reporting hearing loss as a frequent symptom at admission in patients with Ramsay Hunt syndrome [16]. Although no direct anatomical pathway for viral dissemination from the geniculate ganglion to the cochlear nerve, spiral ganglion or cochlea has been described, anatomical studies have demonstrated more extensive nerve fiber interactions that could facilitate viral spread [17].

Taken together, our findings suggest a more direct viral involvement of the neural structures leading to both hearing loss and facial palsy in Ramsay Hunt syndrome.

### 4.1 Limitations

Our sample size was hampered by 36% (25 of 68) of patients not attending follow up as scheduled. Even though not attending follow‐up is likely to reflect a good recovery we cannot speculate if this also includes any detectable emission changes of OAE. In addition, the limited sample size restricts our ability to draw conclusions regarding potential differences between pathogens. Pre‐morbid OAE levels were unknown, and we therefore cannot conclusively attribute the findings on admission to the viral central nervous system infection albeit in patients with significant changes over time, it is reasonable to infer that the initial impairment was related to the viral infection. Furthermore, to minimize this limitation we excluded patients with pre‐existing hearing loss, or a history of significant noise exposure.

The observed changes in OAE levels can be subtle and often too small to be detected clinically or by audiometry even in a young population as patients with viral meningitis. This should favor the use of OAE in patients with CNS infections, especially in patients with decreased level of consciousness which may be the case in patients with encephalitis.

As described in children, congenital viral diseases causing hearing loss is proposed to be due to direct viral invasion of cochlear hair cells whereas hearing loss described in bacterial meningitis is primarily due to inflammatory debris in the cochlea. How this difference can inflict hair cell viability and subsequently influence the mechanics of OAE in other patterns is unknown to us.

### 4.2 Future Perspectives

Given the impairment of the OAE levels in patients with both viral meningitis and Herpes simplex encephalitis, we propose to evaluate if OAE is more prone to be affected in conditions with more disseminated viraemia and may thus serve as a diagnostic adjunct to assist in differentiating between viral meningitis and encephalitis. Also, the objective means of obtaining OAE—no need for patients' response as in audiometry—support OAE in studies aiming to study the pathogenesis of hearing loss and treatments that can protect cochlear function.

## 5 Conclusion

Viral meningitis is associated with outer hair cell dysfunction in the cochlea that can result in sensorineural hearing loss. Hearing outcome in meningitis due to HSV‐2 and VZV virus including the Ramsay Hunt syndrome is good. The broad‐spectrum decrease in OAE levels, reflecting cochlear dysfunction, that is observed in Herpes encephalitis with parenchymal involvement need further clinical evaluation to confirm any clinical use for the separation of viral encephalitis from other viral neuroinfections.

## Author Contributions

Conception and design: ESJ, PCT, MK, and CTB. Data collection: ESJ, JBK, BRH, NH, RW, CTB, and DASGIB Study Group members. Data analysis and interpretation: ESJ, MK, PTP, and CTB. Drafting of article: ESJ, AVJ, and CTB. Article revision: ESJ, PCT, JBK, BRH, NH, RW, MK, CTB, PTP, AVJ, and DASGIB study group. All authors have read and approved the article.

## Ethics Statement

The study did not interfere with patient treatment, and testing with OAE was harmless why no approval was needed according to The Regional Committee on Health Research Ethics in the Capital Region. This study was approved by the Danish Data Protection Agency (2012‐58‐0004, I‐Suite 03637). Written informed consent was obtained from the patients or relatives before enrollment. The Regional Committee on Health Research Ethics in the Capital Region did not require registration, as the study did not interfere with patient treatment, and testing with OAE was harmless (protocol no. H‐1‐2012‐086, author CTB). The study adheres to the Declaration of Helsinki. This study was registered at ClinicalTrials. gov (identifier: NCT03715569).

## Consent

Written informed consent was obtained from the patients or their relatives before enrollment.

## Conflicts of Interest

The authors declare no conflicts of interest.

## Supporting information


Supporting File 1

Supporting File 2

## Data Availability

Due to strict data‐sharing policies in Denmark, we are unable to make our data publicly available. However, researchers who wish to access the data can do so by contacting Professor Christian Brandt (chtb@regionsjaelland.dk) at the Department of Infectious Diseases, Zealand University Hospital or the Regional Data Protection Agency (forskningfortegnelse@regionsjaelland.dk) to obtain permission from the regional data committee.
