# Supplementary material for: Occurrence of Sensorineural Hearing Loss in Viral Meningitis and Encephalitis
Source: J Med Virol. 2026 Oct 5;98(10):e71171. doi: 10.1002/jmv.71171 (PMC13636606; doi:10.1002/jmv.71171)

Enterovirus meningitis

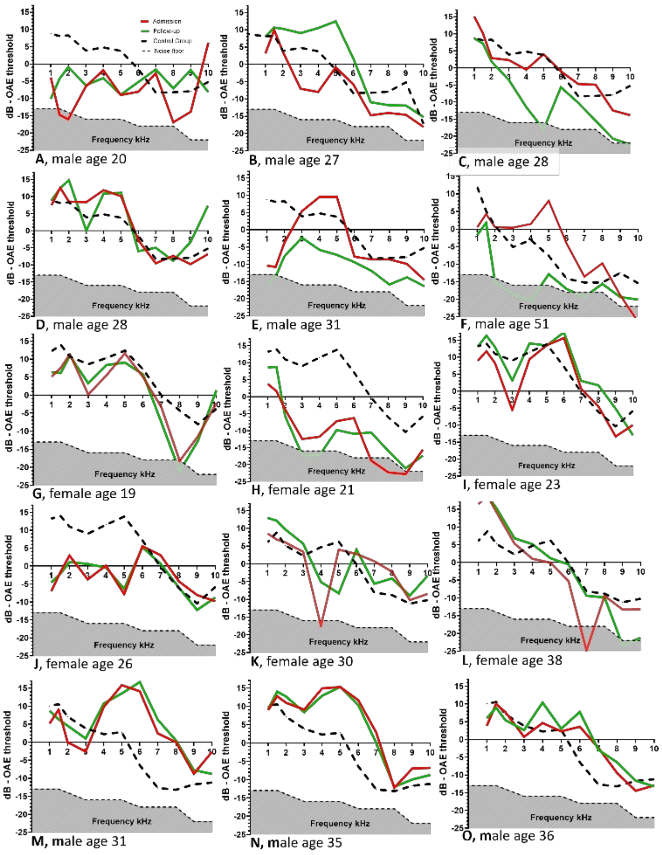

Herpes simplex 2 meningitis

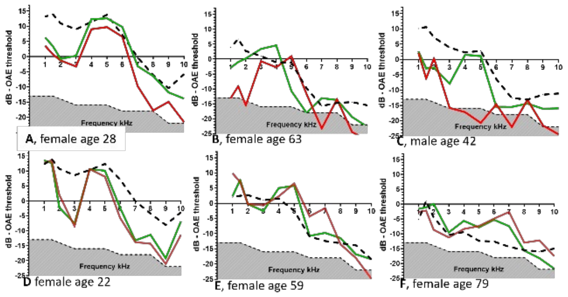

Varicella zoster meningitis

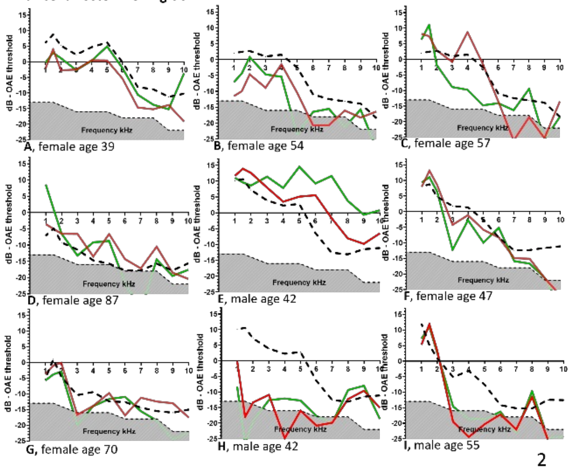

Varicella zoster meningitis with Ramsay Hunt syndrome

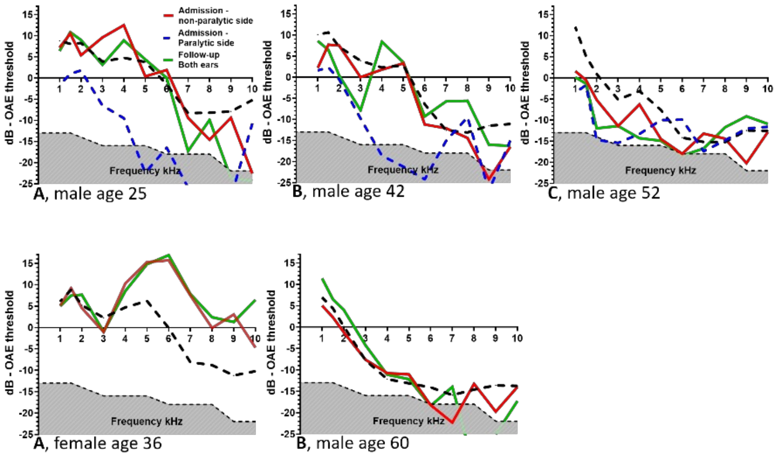

Meningitis with unidentified pathogen

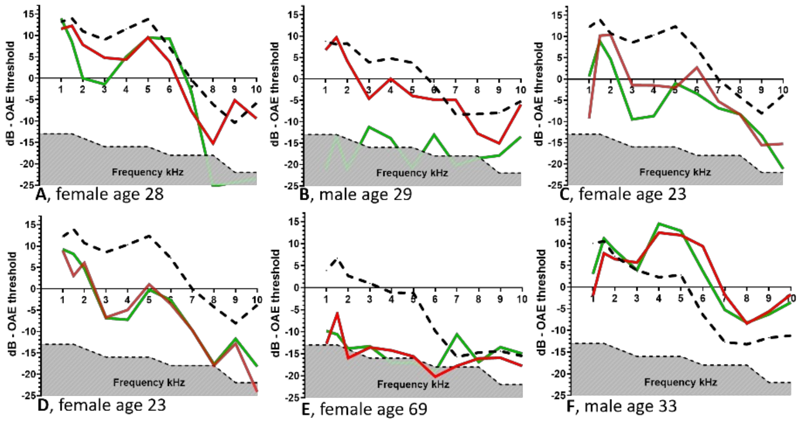

Herpes simplex encephalitis

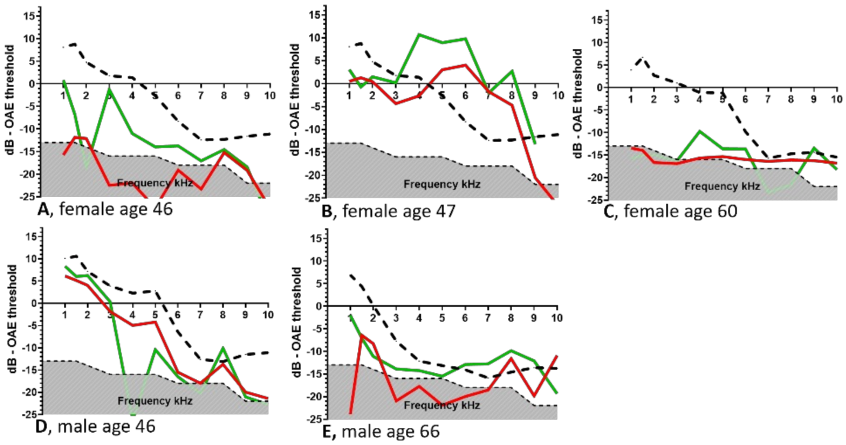

Varicella zoster encephalitis

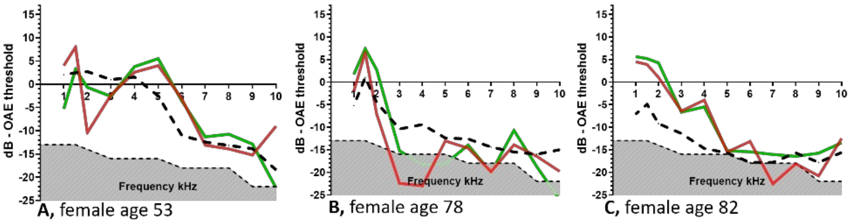

Supplement: Supplementary file 1 [file JMV-98-e71171-s001.docx]
