# Supplementary material for: Occurrence of Sensorineural Hearing Loss in Viral Meningitis and Encephalitis
Source: J Med Virol. 2026 Oct 5;98(10):e71171. doi: 10.1002/jmv.71171 (PMC13636606; doi:10.1002/jmv.71171)
Supplement: Supplementary file 2 [file JMV-98-e71171-s002.docx]

**Supplemental figure 1: OAE levels in dB in 11 frequencies** (1, 1.5, 2, 3, 4, 5, 6, 7, 8, 9 and 10▒kHz). OAE levels on admission (**RED** line), follow-up (**GREEN** line) and paralytic side in Ramsay Hunt syndrome (**BLUE** dotted line) in all included patients with viral meningitis and encephalitis. The **thick dotted line** show the mean OAE levels in an age- and sex matched control group. The **dotted thin line with dark grey area below** delineates the noise floor.

**Enterovirus meningitis:** Patients A, B and D had increase of OAE levels and C, E and F had decrease. Patients G to O did not have OAE changes in any frequency category >5▒dB.

**Herpes simplex 2 meningitis:** Patients A, B and C had recovery of OAE. Patients D, E and F had no changes in any frequency category >5▒dB.

**Varicella Zoster meningitis:** Patient A, B, D and E had increase in OAE levels. Patient C had decreased OAE levels. Patient F to I had no changes in any frequency category >5▒dB.

**Varicella Zoster meningitis with Ramsay Hunt syndrome:** Patient A, B had increase in OAE levels on facial paralytic side. Patient C had similarly low OAE levels on the paralytic side but without significant increase from admission to follow-up.

**Other Pathogen:** Patients A and B had no changes >5▒dB.

**Meningitis with Unidentified pathogen:** Patient A and B had a decrease of OAE levels. Patients C to F had no changes in any frequency category >5▒dB.

**Herpes simplex encephalitis:** Patient A and B had increase and patient D a decrease of OAE levels. Patient C and E had no changes in any frequency category >5▒dB.

**Varicella zoster encephalitis:** Patients A, B and had no changes >5▒dB.
